# Antimicrobial resistance genes in a golden jackal (*Canis aureus* L. 1758) from Central Italy

**DOI:** 10.1007/s11259-023-10172-4

**Published:** 2023-07-12

**Authors:** A. Di Francesco, D. Salvatore, M. Gobbi, B. Morandi

**Affiliations:** 1 Department of Veterinary Medical Sciences (DIMEVET), University of Bologna, Ozzano dell’ Emilia (BO), Italy; 2 Istituto Zooprofilattico Sperimentale dell’Umbria e delle Marche ‘Togo Rosati’, Perugia, Italy

**Keywords:** Antimicrobial resistance genes, Golden jackal, *Canis aureus*, Italy, One health, Wildlife

## Abstract

In recent years an increasing interest has been focused on the contribution of wildlife in ecology and evolution of the antimicrobial resistance (AMR). The aim of this study was to molecularly investigate the presence of antimicrobial resistance genes (ARGs) in organ samples from a golden jackal (*Canis aureus*) found dead in the Marche region (Central Italy). Samples from lung, liver, spleen, kidney, and intestine were investigated by PCRs targeting the following genes: *tet*(A), *tet*(B), *tet*(C), *tet*(D), *tet*(E), *tet*(G), *tet*(K), *tet*(L), *tet*(M), *tet*(O), *tet*(S), *tet*(P), *tet*(Q), *tet*(X), *sul1*, *sul2*, *sul3*, *bla*_CTX−M_, *bla*_SHV_, *bla*_TEM_, and *mcr*-1 to *mcr*-10. One or more ARGs were detected in all organs tested, except the spleen. Specifically, the lung and liver were positive for *tet*(M) and *tet*(P), the kidney for *mcr*-1 and the intestine for *tet*(A), *tet*(L), *tet*(M), *tet*(O), *tet*(P), *sul3* and *bla*_TEM−1_. These results, according to the opportunistic foraging strategy of the jackal, confirm its potential role as a good bioindicator of AMR environmental contamination.

## Introduction

Antimicrobial resistance (AMR) in natural ecosystems is of increasing concern to public and animal health (Arnold et al. 2016). Usually, wild animals are supposed to be less likely exposed to clinical antimicrobial agents than domestic animals or humans, except, occasionally, in rehabilitation facilities (Plaza-Rodriguez et al. 2021). However, antimicrobial resistant bacteria and/or antimicrobial resistance genes (ARGs) have been highlighted in numerous wildlife species (Ramey 2021). This phenomenon could be attributed to the natural production of antimicrobial molecules by strains of bacteria and fungi in all environments, including soil (Allen et al. 2010). However, AMR in wildlife cannot be considered an exclusively natural phenomenon since multi-drug resistant strains were more frequently found in wild animals from more anthropized sites, as a consequence of anthropic AMR pollution of the ecosystems they inhabit (Laborda et al. 2022). Indeed, wildlife is an integral part of the natural environmental compartment and can be influenced by anthropogenic contaminations (e.g., human waste-management practices and/or animal husbandry facilities) which can be a source of active antimicrobials, antimicrobial-resistant bacteria, and ARGs. Therefore, increasing interest has turned to wild animals in order to evaluate their role as environmental reservoirs/vectors of antimicrobial resistant bacteria or bioindicators of AMR pollution (Laborda et al. 2022).

Conventionally, AMR is investigated using culture-dependent methods based on bacteriological culture and antibiotic susceptibility testing of isolated microorganisms. Recently, a molecular approach based on amplification of AMR target genes from environmental or biological samples has been proposed (Galhano et al. 2021). This approach is more expensive than traditional cultivation and since it does not allow the determination of the bacterial sources of the ARGs detected it can be used as an epidemiological but non-diagnostic tool. However, it is a rapid method that avoids possible AMR underestimation resulting from non-culturable or slow-growing bacteria, proving to be particularly useful in a context such as wildlife, where the advanced state of decomposition of the carcasses often reduces the reliability of culture-dependent methods. The aim of this study was to investigate, by PCR, the presence of ARGs in organ samples from a golden jackal (*Canis aureus*).

## Materials and methods

In March 2023 the carcass of a golden jackal was recovered in the municipality of Porto Potenza (43°21’29.99’’N; 13°39’10.18’’E), province of Macerata (Marche region, Central Italy) and then submitted to the Istituto Zooprofilattico Sperimentale Umbria and Marche ‘Togo Rosati’ of Tolentino (Marche region), to determine the cause of death. During necropsy, which highlighted several wounds attributable to bites from other canids, samples from lung, liver, spleen, kidney, and intestine (jejunum) were collected, stored at − 20 °C and sent to Department of Veterinary Medical Sciences (Ozzano dell’Emilia, Bologna, Italy) for molecular investigations.

Total DNA was extracted from each organ sample using the QIAamp DNA mini kit (Qiagen, Hilden, Germany) following the supplier’s recommendations. One extraction control, consisting of kit reagents only, was included. DNA samples were investigated for the presence of ARGs against antimicrobials extensively used both in human and veterinary fields, as tetracyclines, sulphonamides, β-lactams and colistin. Specifically, the following clinically relevant genes were tested: *tet*(A), *tet*(B), *tet*(C), *tet*(D), *tet*(E), *tet*(G), *tet*(K), *tet*(L), *tet*(M), *tet*(O), *tet*(S), *tet*(P), *tet*(Q), *tet*(X), *sul1*, *sul2*, *sul3*, *bla*_CTX−M_, *bla*_SHV_, *bla*_TEM_, and *mcr*-1 to *mcr*-10. Each gene was amplified by an individual PCR, using primers according to Ng et al. 2001 (*tet* genes), Sáenz et al. 2004 (*sul* genes), Batchelor et al. 2005 (*bla*_CTX−M_), Jouini et al. 2007 (*bla*_SHV_ and *bla*_TEM_), Liu et al. 2016 (*mcr*-1), Zhang et al. 2018 (*mcr*-2 to *mcr*-5), Gorecki et al. 2022 (*mcr*-6 to *mcr*-9) and Lei et al. 2020 (*mcr*-10). The following PCR protocols were carried out: 5 min of initial denaturation at 94 °C followed by 35 cycles at 94 °C for 1 min, 50 °C [*tet*(K)], 51 °C [*tet*(P), *tet*(S) and *sul3*], 51.3 °C (*mcr*-8 and *mcr*-9), 53 °C [*tet*(B), *tet*(D), *tet*(E), *tet*(M), *tet*(Q), *tet*(X), *bla*_SHV_ and *mcr*-1], 55 °C [*tet*(A), *tet*(C), *tet*(G), *tet*(L), *tet*(O), *sul2*, *bla*_CTX−M_ and *bla*_TEM_], 56 °C (*mcr*-7), 57 °C (*mcr*-2, *mcr*-3, *mcr*-4, *mcr*-6 and *mcr*-10), 59 °C (*sul1*) or 62 °C (*mcr*-5) for 1 min, and 72 °C for 1 min. A final extension step of 10 min at 72 °C completed the reaction. The DNA extracted from more *Escherichia coli* strains containing antimicrobial resistance plasmids, was used as a positive control. The extraction control and a distilled water negative control were also included. The PCR products were analysed by 2% agarose gel electrophoresis; the DNA bands were stained with Midori Green Advance (Nippon Genetics Europe GmbH, Düren, Germany) and then visualised using ultraviolet (UV) trans illumination. The amplicons were purified using the High Pure PCR Product Purification Kit (Roche, Mannheim, Germany) and both DNA strands were sequenced (Bio-Fab Research, Rome, Italy). The sequences obtained were compared with the public sequences available using the BLAST server in the GenBank database (National Center for Biotechnology Information 2023).

## Results and discussion

The results are shown in Table 1.

**Table 1:** Antimicrobial resistance genes detected in the samples tested

Detected genes	Lung	Liver	Spleen	Kidney	Intestine
*tet*(A)	−	−	−	−	**+**
*tet*(L)	−	−	−	−	**+**
*tet*(M)	**+**	**+**	−	−	**+**
*tet*(O)	−	−	−	−	**+**
*tet*(P)	**+**	**+**	−	−	**+**
*sul3*	−	−	−	−	**+**
*bla*_TEM−1_	−	−	−	−	**+**
*mcr*-1	−	−	−	**+**	−

The identity of the amplicons was confirmed by comparison between the sequences obtained and the corresponding sequences available in the GenBank database, showing 99–100% nucleotide similarity. A representative sequence for each amplified gene was deposited in the GenBank database under accession numbers OR026616-OR026623.

The ARGs targeted in this study were chosen on the basis of the diffusion and/or emergence of the AMR they are responsible for.

Tetracyclines are broad-spectrum antibacterial agents, which show activity against most Gram-positive and Gram-negative bacteria, both anaerobic and aerobic. The combination of broad-spectrum activity and low toxicity has led to their intensive use in human and animal infection therapy including as growth promoters in food animal production systems, favouring the widespread tetracycline resistance phenomenon. Tetracycline resistance is generally caused by the acquisition of tetracycline resistance (*tet*) genes, often associated with either a mobile plasmid or a transposon. Three main resistance mechanisms are mediated by *tet* genes: pumping the drug out of the cell before it reaches its site of action (active efflux pumps), protection of the ribosomal binding site which decreases drug binding, and enzymatic inactivation of the active compound. The first two mechanisms currently predominate in clinical settings (Roberts 2005). Regarding to the 14 tetracycline resistance genes tested, five *tet* genes were detected: *tet*(A), *tet*(L) and *tet*(P), involved in active efflux pumps and *tet*(M) and *tet*(O), responsible for protection of the ribosomal binding site. The most represented *tet* genes in the samples tested were *tet*(M) and *tet*(P). With respect to *tet*(M), it has been detected in at least 81 different bacterial genera, probably because of its association with conjugative transposons that appear to have lower host specificity than plasmids. More limited taxonomic distribution has been assigned to *tet*(P) gene, highlighted in only 3 bacterial genera (https://faculty.washington.edu/marilynr/tetweb2.pdf, https://faculty.washington.edu/marilynr/tetweb3.pdf).

Sulphonamides were the first drugs to be used in veterinary medicine in therapeutic doses. Their excessive usage imposed widespread selective pressures on bacteria, as seen by the high prevalence rates of sulphonamide resistance observed in mainly Gram-negative bacteria isolated from animals and humans (Pavelquesi et al. 2021). Resistance to sulphonamides is associated with the presence of *sul* genes located in transposons and in self-transferable or mobilizable plasmids and encoding a variant of the dihydropteroate synthetase enzyme with a low affinity for sulphonamides. In this study only *sul3* was detected, although the literature reported a higher frequency of *sul1* and *sul2* (Wang et al. 2014).

β-lactams are one of the most commonly prescribed drug classes with numerous clinical indications. Resistance to β-lactams, resulting from their use and overuse, is primarily due to bacterially produced β-lactamase enzymes that hydrolyse the β-lactam ring, rendering the antibiotic ineffective. Resistance to the β-lactams continues to increase, especially in Gram-negative organisms, posing a public health concern exacerbated by the rapid evolution of extended-spectrum β-lactamases (ESBLs) which are a group of enzymes that confer resistance to most β-lactam antibiotics, including expanded-spectrum cephalosporins and monobactams (Castanheira et al. 2021). Many different types of ESBLs have been described up until now. However, the most common ones are variants of the CTX-M, SHV and TEM enzymes, due to amino acid substitutions leading to a change in their substrate profile. With regard to the *bla*_TEM−1_ gene, the first TEM allele identified, it confers resistance to penicillins, and early cephalosporins; this gene needs only a few specific single nucleotide polymorphisms to evolve into a gene encoding an extended-spectrum β-lactamase (Muhammad et al. 2014). In this study the sequence analysis of the *bla*_TEM_ amplicon obtained did not highlight the amino acid residues that are most frequently involved in conferring the ESBL phenotype to TEM-type enzymes (Bradford 2001). The comparison with the corresponding sequences available in the GenBank database allowed the amplicon to be identified as *bla*_TEM−1_.

Colistin is a polymyxin antibiotic that has been used in veterinary medicine for decades, as a treatment for enterobacterial digestive infections as well as a prophylactic treatment and growth promoter in livestock animals, leading to the emergence and spread of colistin-resistant Gram-negative bacteria. The colistin resistance represents a great public health concern, considering that colistin is one of the last-resort antibiotics against multidrug-resistant deadly infections, in particular by strains resistant to carbapenems (Valiakos and Kapna 2021). With respect to the 10 colistin resistance genes tested, only *mcr*-1, which is the most commonly encountered one, was highlighted.

One or more AMR genes were detected in all organs tested, except the spleen. In particular, the lung and liver were positive for *tet*(M) and *tet*(P), the kidney for *mcr*-1 and the intestine for *tet*(A), *tet*(L), *tet*(M), *tet*(O), *tet*(P), *sul3* and *bla*_TEM−1_. The high percentage of positivity highlighted in the intestine could be attributed to frequent finding of ARGs associated with commensal gut microbiota (Arnold et al. 2016). However, the detection of ARGs from lung, liver and kidney suggests their association with bacteria responsible for infection. Cross-contaminations could be excluded because only intact organs were examined, and the DNA extraction was performed on internal fragments of each organ that had been removed with a disposable scalpel blade. Confirming this, *tet*(A), *tet*(L), *tet*(O), *sul3* and *bla*_TEM−1_ genes were detected only in the intestine and the *mcr*-1 gene only in the kidney.

The sampled golden jackal is the first official sighting of *Canis aureus* in the Marche region (Central Italy), evidently due to an expansion of its range after entering Italy in the mid-1980s from the Balkans via Friuli Venezia Giulia (Lapini et al. 2011). Since then, golden jackals have gradually been making their way south through Italy. To date, the literature related to AMR in *Canis aureus* is scarce (Namroodi et al. 2017). In the absence of data on AMR in golden jackal, studies on the red fox (*Vulpes vulpes*), a generalist and opportunistic carnivore against which the jackal often assumes a dominant position, could be taken as a reference. The literature on red fox showed a high diversity and number of ARGs conferring resistance mainly to aminoglycosides, tetracyclines, macrolide-lincosamide-streptogramin B and β-lactams (Dias et al. 2022); these findings could be related to the increasing abundance of foxes in urban and peri-urban areas.

Similarly, the finding of ARGs in a golden jackal is not surprising considering the opportunistic feeding habits which allowed it to profit from anthropogenic food sources. Although the golden jackal was found to eat mainly small mammals, such as rodents (54% biomass), its diet also includes domestic animal and ungulate waste from slaughtering and hunting or plants when other meat is not readily available (Lange et al. 2021). In this regard, the necropsy of the golden jackal sampled in this study detected the presence of sheep fleece and chicken feathers in the stomach.

The flexibility of food sources, some of which are anthropogenic, the high position in the food chain and the ability to survive in different contexts make the jackal a good bioindicator of environmental contamination. In this regard, the golden jackal has been proposed as a biological indicator of the presence of heavy metals in the sites which it inhabits, as it has been demonstrated to have a high potential for their specific accumulation (Markov et al. 2016). Interestingly, recent studies (Gupta et al. 2022) highlighted that sites with high concentrations of heavy metal from agricultural wastes, mining and industrial releases, urban runoff, treated and untreated domestic wastes, and livestock farming, had a higher level of specific bacterial hosts carrying ARGs, suggesting that antibiotics may not be the only source of environmental selective pressure on the bacterial communities.

In conclusion, the results of this study highlight a potential role of *Canis aureus* as a good bioindicator and/or vector of AMR environmental contamination, suggesting further insights into a larger sample of animals. In addition, the detection of ARGs on biological samples by molecular methods seems to be an effective investigative tool in studying the dynamics of AMR diffusion in the environment.

## Data Availability

All data used and/or analysed during this study are included in this article and are available from the corresponding author on reasonable request. The sequences generated in this study are available in GenBank under accession numbers OR026616-OR026623.
